# Oral microbiome sequencing revealed the enrichment of *Fusobacterium* sp., *Porphyromonas* sp., *Campylobacter* sp., and *Neisseria* sp. on the oral malignant fibroma surface of giant panda

**DOI:** 10.3389/fcimb.2024.1356907

**Published:** 2024-05-28

**Authors:** Xiaowan Wang, Meiling Jing, Qizhao Ma, Yongwang Lin, Ting Zheng, Jiangchuan Yan, Libing Yun, Chengdong Wang, Yuqing Li

**Affiliations:** ^1^ State Key Laboratory of Oral Diseases, National Center for Stomatology, National Clinical Research Center for Oral Diseases, West China Hospital of Stomatology, Sichuan University, Chengdu, Sichuan, China; ^2^ Department of Forensic Pathology, West China School of Basic Medical Sciences and Forensic Science, Sichuan University, Chengdu, Sichuan, China; ^3^ China Conservation and Research Centre for the Giant Panda, Key Laboratory of SFGA on The Giant Panda, Chengdu, Sichuan, China

**Keywords:** giant panda, oral tumor, 16S rRNA gene sequencing, oral malignant fibroma, *Fusobacterium*, *Porphyromonas*

## Abstract

**Introduction:**

Microbial community composition is closely associated with host disease onset and progression, underscoring the importance of understanding host–microbiota dynamics in various health contexts.

**Methods:**

In this study, we utilized full-length 16S rRNA gene sequencing to conduct species-level identification of the microorganisms in the oral cavity of a giant panda (*Ailuropoda melanoleuca*) with oral malignant fibroma.

**Results:**

We observed a significant difference between the microbial community of the tumor side and non-tumor side of the oral cavity of the giant panda, with the latter exhibiting higher microbial diversity. The tumor side was dominated by specific microorganisms, such as *Fusobacterium simiae*, *Porphyromonas* sp. feline oral taxon 110, *Campylobacter* sp. feline oral taxon 100, and *Neisseria* sp. feline oral taxon 078, that have been reported to be associated with tumorigenic processes and periodontal diseases in other organisms. According to the linear discriminant analysis effect size analysis, more than 9 distinct biomarkers were obtained between the tumor side and non-tumor side samples. Furthermore, the Kyoto Encyclopedia of Genes and Genomes analysis revealed that the oral microbiota of the giant panda was significantly associated with genetic information processing and metabolism, particularly cofactor and vitamin, amino acid, and carbohydrate metabolism. Furthermore, a significant bacterial invasion of epithelial cells was predicted in the tumor side.

**Discussion:**

This study provides crucial insights into the association between oral microbiota and oral tumors in giant pandas and offers potential biomarkers that may guide future health assessments and preventive strategies for captive and aging giant pandas.

## Introduction

The giant panda (*Ailuropoda melanoleuca*) was classified as a vulnerable species by the International Union for Conservation of Nature in 2016 and has thus been under rigorous protection in China (Wei et al., 2020). They are renowned for having a predominantly bamboo-based diet and can spend over 14 hours a day consuming more than 30 pounds of bamboo to fulfill their energy requirements (Viswanathan, 2010). Several studies found that oral diseases in giant pandas not only disrupt their feeding but, in certain cases, lead to their mortality (Jin et al., 2015). Additionally, oral tumors present significant challenges to the well-being of giant pandas, especially older individuals who, due to dental degradation or loss, have specific dietary needs. Therefore, exploring the mechanisms of oral carcinogenesis and minimizing the incidence of oral tumors is paramount for the conservation of giant pandas.

The oral cavity of giant pandas is a complex, warm, and moist environment, similar to that of humans. Various factors, including diet, age, and living conditions, can contribute to the development of oral diseases. Although, in recent years, extensive research has been conducted on oral diseases, such as dental caries (Liu et al., 2020), periodontitis (Reyes, 2021), and oral tumors (Chen et al., 2021), in humans and animals, there are limited reports on oral diseases in giant pandas. Increasing evidence underscores the intrinsic relationship between oral diseases and resident microorganisms. Additionally, some studies suggest a strong association between oral tumors and oral microbial species (El Tekle and Garrett, 2023; Hayes et al., 2018; Stasiewicz and Karpinski, 2022), including *Peptostreptococcus oralis*, *Streptococcus salivarius*, and *Streptococcus grigneri* (Pushalkar et al., 2012; Zhang et al., 2020). A previous study elucidated the role of the oral microbiome in the pathogenesis, progression, and metastasis of oral tumors in humans (Li et al., 2023). While some studies have delved into the oral microbe-disease nexus in pets, like cats and dogs (Davis and Weese, 2022), research on the oral microbiome of giant pandas is limited.

Jin isolated a total of 253 bacterial strains, representing 23 genera and 48 species, from the oral cavity of giant pandas (Jin et al., 2012). Among these, the predominant bacterial genera included *Streptococcus*, *Moraxella*, *Peptostreptococcus*, and *Porphyromonas*. Additionally, a comprehensive analysis of the caries-related microbiome in giant pandas detected 268 bacterial species, spanning 189 genera, 98 families (Ma et al., 2022). Among these, the dominant genera included unclassified *Neisseriaceae*, *Actinobacillus*, *Lautropia*, *Neisseria*, *Porhyromonas*, unclassified *Pasteurellaceae*, *Moraxella*, *Streptococcus*, *Bergeywlla*, and *Capnocytophaga*. The paucity of research concerning the interplay between oral microorganisms and the incidence of oral diseases in giant pandas has been particularly underscored.

In this study, we aimed to explore the association between oral malignant fibroma and the oral microbiome of giant pandas by collecting oral samples from an afflicted individual and conducting the full-length 16S rRNA gene sequencing analysis by PacBio technology. Our aim is to identify the microorganism composition and abundance that may be linked to the occurrence of oral tumors in giant pandas, with the ultimate goal of providing guidance for safeguarding their oral and overall health.

## Materials and methods

### Samples

In this study, we examined a 23-year-old elderly female giant panda with a tumor located on the right side of her oral cavity. The tumor originated from the right buccal mucosa and measured 9.2 cm × 5.0 cm, as visualized on a computed tomography scan. Immunohistochemical results of the tumor were, AE1/AE3 (-), CK (-), EMA (-), S100 (+), SMA (-), CD34 (-), CD31 (-), Desmin (-), Vimentin (+ +), Calponin (-), CD99 (-), Ki67 (positive rate about 1%), and the immunophenotype and biological behavior were indicative of a low-grade malignant fibroma. Microbial sampling from various niches (mucosa, dental plaque, tumor surface, and vestibular sulcus) within the oral cavity were collected using sterile cotton swabs. Oral hygiene and surgical removal of the tumor were performed after sampling. We obtained a total of 16 samples, whose names and abbreviations are provided in **Table 1**. The samples were immediately stored in sterile phosphate-buffered saline on dry ice and kept at −80°C for further analysis. The samples were collected in June 2022 at the Dujiangyan base of the China Conservation and Research Center for the Giant Panda in Chengdu, Sichuan province. All animal experiments were approved by Animal Care and Use Committee of the China Conservation and Research Center (Letter no: CCRCGP2020003).

**Table 1:** Site niches and abbreviations for sample sampling and grouping method are indicated.

	Name	Abbreviation		Name	Abbreviation
Tumor side	Upper Right Buccal Mucosa	URBMuc	Non-tumor side	Upper Left Buccal Mucosa	ULBMuc
	Lower Right Buccal Mucosa	LRBMuc		Lower Left Buccal Mucosa	LLBMuc
	Upper Right Canine	URCan		Upper Left Canine	ULCan
	Lower Right Canine	LRCan		Lower Left Canine	LLCan
	Upper Right Molar	URMol		Upper Left Molar	ULMol
	Lower Right Molar	LRMol		Lower Left Molar	LLMol
	Right Vestibular Sulcus	RVesSul		Left Vestibular Sulcus	LVesSul
	Surface of Tumor	STum			
	Periphery of Tumor	PTum			

### DNA extraction

Total genomic DNA samples were extracted using the OMEGA Soil DNA Kit (M5635-02; Omega Bio-Tek, Norcross, GA, USA), following the manufacturer’s instructions, and stored at −20°C prior to further analysis. The quantity and quality of the extracted DNA samples were measured using the NanoDrop NC2000 spectrophotometer (Thermo Fisher Scientific, Waltham, MA, USA) and agarose gel electrophoresis, respectively.

### 16S rRNA gene sequencing

Polymerase chain reaction (PCR) of the full-length bacterial 16S rRNA genes was performed using the forward primer 27F (5’-AGAGTTTGATCMTGGCTCAG-3’) and the reverse primer 1492R (5’-ACCTTGTTACGACTT-3’). Thereafter, sample-specific 16-bp barcodes were incorporated into the primers for multiplex sequencing in the second step of PCR. Each PCR sample contained 5 μL of buffer (5×), 5 μL of GC buffer (5×), 0.25 μL of Q5 DNA polymerase (5 U/μL), 2 μL (2.5 mM) of dNTPs, 1 μL (10 μM) each of forward and reverse primers, 2 μL of DNA template, and 8.75 μL of ddH_2_O. PCR was conducted under the following conditions: initial denaturation at 98°C for 2 min; followed by 25/10 cycles (for first and second PCR amplification steps, respectively) of denaturation at 98°C for 30 s, annealing at 55°C for 30 s, and extension at 72°C for 90 s; and final extension at 72°C for 5 min. The PCR amplicons were purified using Agencourt AMPure Beads (Beckman Coulter, Indianapolis, IN) and quantified using the PicoGreen dsDNA Assay Kit (Invitrogen, Carlsbad, CA, USA). Subsequently, the amplicons were pooled in equal amounts and subjected to single-molecule real-time sequencing using the PacBio Sequel platform at Shanghai Personal Biotechnology Co., Ltd (Shanghai, China). PacBio circular consensus sequencing (CCS) reads were generated from multiple alignments of sub-reads to reduce sequencing errors. In CCS, DNA polymerase reads a ligated circular DNA template multiple times to generate a consensus sequence from multiple reads of a single molecule. The raw sequences were filtered for a minimum of 3 passes through the PacBio SMRT Link portal (version 5.0.1.9585) until a minimum predicted accuracy of 99% was achieved. The predicted accuracy threshold was defined as the level below which CCS acted as noise. Files generated by the PacBio platform were trimmed to < 2,000 bp amplicon size.

### Sequence analysis

Bioinformatics was conducted using QIIME2 2022.11 (Bolyen et al., 2019) with slight modification. Briefly, raw sequence data were subjected to demultiplexing using the demux plugin followed by primer cutting using the cutadapt plugin. Sequences were then merged, quality filtered, and dereplicated using the fastq-mergepairs, fastq-filter, and derep-fullength functions, respectively, in the Vsearch plugin. Non-singleton amplicon sequence variants (ASVs) were aligned with mafft (Katoh et al., 2002) and used to construct a phylogeny with fasttree2 (Price et al., 2009). Alpha- and beta-diversity metrics were estimated using the diversity plugin with samples rarefied to 989 sequences per sample. Taxonomy was assigned to ASVs using the classify-sklearn naïve Bayes taxonomy classifier in the feature-classifier plugin (Bokulich et al., 2018) against the SILVA Release 132 database (Koljalg et al., 2013). The raw reads were submitted to the NCBI Sequence Read Archive (SRA) database with the assigned Accession Number: PRJNA1053871.

### Bioinformatics and statistical analysis

Sequence data analyses were performed using the QIIME2 and R packages (v3.2.0). ASV-level alpha diversity indices, such as the Chao1 richness estimator (Chao, 1984), Observed species, Shannon diversity index (Shannon, 1948a; Shannon, 1948b), Simpson index (Simpson, 1949), Pielou’s evenness (Pielou, 1966), and Good’s coverage were calculated using the ASV table in QIIME2 and visualized as box plots. ASV-level ranked abundance curves were generated to compare the richness and evenness of the ASVs among samples. Beta diversity analysis was performed to investigate the structural variation of microbial communities across samples using Bray-Curtis metrics (Bray and Curtis, 1957) and visualized via principal coordinate analysis (PCoA) and unweighted pair-group method with arithmetic means (UPGMA) hierarchical clustering (Ramette, 2007). Significant variations in the microbiota structure among the groups were assessed by permutational multivariate analysis of variance (PERMANOVA) (McArdle and Anderson, 2001), analysis of similarities (Clarke, 1993; Warton et al., 2012), and permutation test of multivariate homogeneity of groups dispersions (Anderson et al., 2006) using QIIME2. The microbiota composition and abundance were visualized using MEGAN (Huson et al., 2011) and GraPhlAn (Asnicar et al., 2015). Venn diagram was generated using the R package “VennDiagram” to visualize the shared and unique ASVs among groups, based on their occurrence, regardless of their relative abundance (Zaura et al., 2009). Linear discriminant analysis effect size (LEfSe) was performed to detect differentially abundant taxa across groups using the default parameters (Segata et al., 2011). Microbial functions were predicted by phylogenetic investigation of communities by reconstruction of unobserved states (PICRUSt2) (Douglas et al., 2020) using the Kyoto Encyclopedia of Genes and Genomes (KEGG) (https://www.kegg.jp/) database.

## Results

### Overall sequence statistics

To conduct a comparative analysis of the oral microbiota, the samples obtained from the right and left regions were classified as tumor side and non-tumor side samples, respectively. The samples included in this study are enumerated in **Table 1**. A total of 269,344 sequences were acquired (16,834/sample), which were merged, quality filtered, and dereplicated using the Vsearch plugin, to obtain 200,817 ASVs (12,551/sample). To achieve consistent sequencing depth across samples, the sequence data for each sample was extracted using the rarefaction method, resulting in 989 ASVs per sample. Rarefaction analysis was conducted to confirm the validity of the sequencing data. The rarefaction curves for all the samples demonstrated a plateau, indicating that the sequencing depth was adequate to capture the majority of gene diversity (**Supplementary Figure S1**). The distribution of sequenced lengths ranged between 572–1899 bp, with the majority of sequences (n = 23,230) being 1463 bp (**Supplementary Figure S2**).

### Taxonomic composition analysis

**Figure 1:**
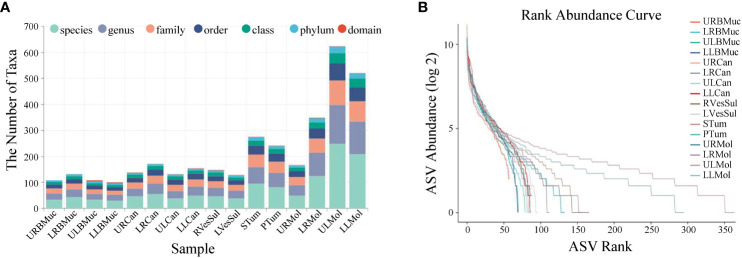
Abundance statistics of microbial species in the oral cavity of the giant panda with an oral tumor. **(A)** The number of samples at various taxonomic levels. **(B)** Rank abundance curves. Dashed lines of different colors correspond to distinct samples. The x-axis denotes the sequential order of amplicon sequence variants (ASVs) sorted by abundance and the y-axis represents the abundance value of each ASV in the sample after the log 2 transformation. The length of the dashed line on the horizontal axis indicates the abundance of the ASVs in the sample. The gradient of the dashed line reflects the evenness of the community composition. A flatter line indicates a more even community composition with a smaller disparity in abundance among ASVs, while a steeper line indicates a less even community composition.

Taxonomic richness varied at the different levels across all samples, resulting in the identification of 31 phyla, 55 classes, 89 orders, 136 families, 219 genera, and 402 species (**Figure 1A**). At the species level, the upper left molar and lower left molar samples exhibited the highest abundance, with 248 and 209 species, respectively. In contrast, the species content of the lower left buccal mucosa was the lowest, amounting to only 30 species. According to the abundance ranking curve (**Figure 1B**), the microbial composition of the left dental plaque appears to be relatively uniform, while those of the other regions appear to be uneven, as observed by the steep slope of the curves.

The top 20 abundant species identified in our study were *Ottowia* sp. canine oral taxon 014, *Spodiobacter cordis*, *Porphyromonas* sp. feline oral taxon 110, *Moraxella* sp. canine oral taxon 396, *Neisseria shayeganii*, *Capnocytophaga* sp. H4358, *Fusobacterium simiae*, *Neisseria* sp. feline oral taxon 145, *Xanthomonadaceae* bacterium feline oral taxon 091, *Aggregatibacter aphrophilus*, *Lautropia* sp. canine oral taxon 060, *Globicatella* sp. feline oral taxon 122, *Glaesserella parasuis*, *Cardiobacterium* sp. canine oral taxon 177, *Neisseria* sp. feline oral taxon 078, *Leptotrichia* sp. canine oral taxon 345, *Neisseria* sp. VA252/2008, *Campylobacter* sp. feline oral taxon 100, *Streptococcus parasuis*, and *Streptococcus minor* (**Supplementary Table S1**).

Owing to the complex and diverse nature of the oral microbiota composition of the giant panda, we conducted a comprehensive detection of its microbial species. The results revealed the presence of *Ottowia* sp. canine oral taxon 014, *S. cordis*, *Moraxella* sp. canine oral taxon 396, *N. shayeganii*, *Capnocytophaga* sp. H4358, *Xanthomonadaceae* bacterium feline oral taxon 091, *A. aphrophilus*, *Lautropia* sp. canine oral taxon 060, and *Cardiobacterium* sp. canine oral taxon 177 in various niches of the oral cavity. However, the other species were confined to specific regions within the oral cavity, suggesting a complex and diverse microbial composition in these areas.

### Alpha diversity analysis

**Figure 2:**
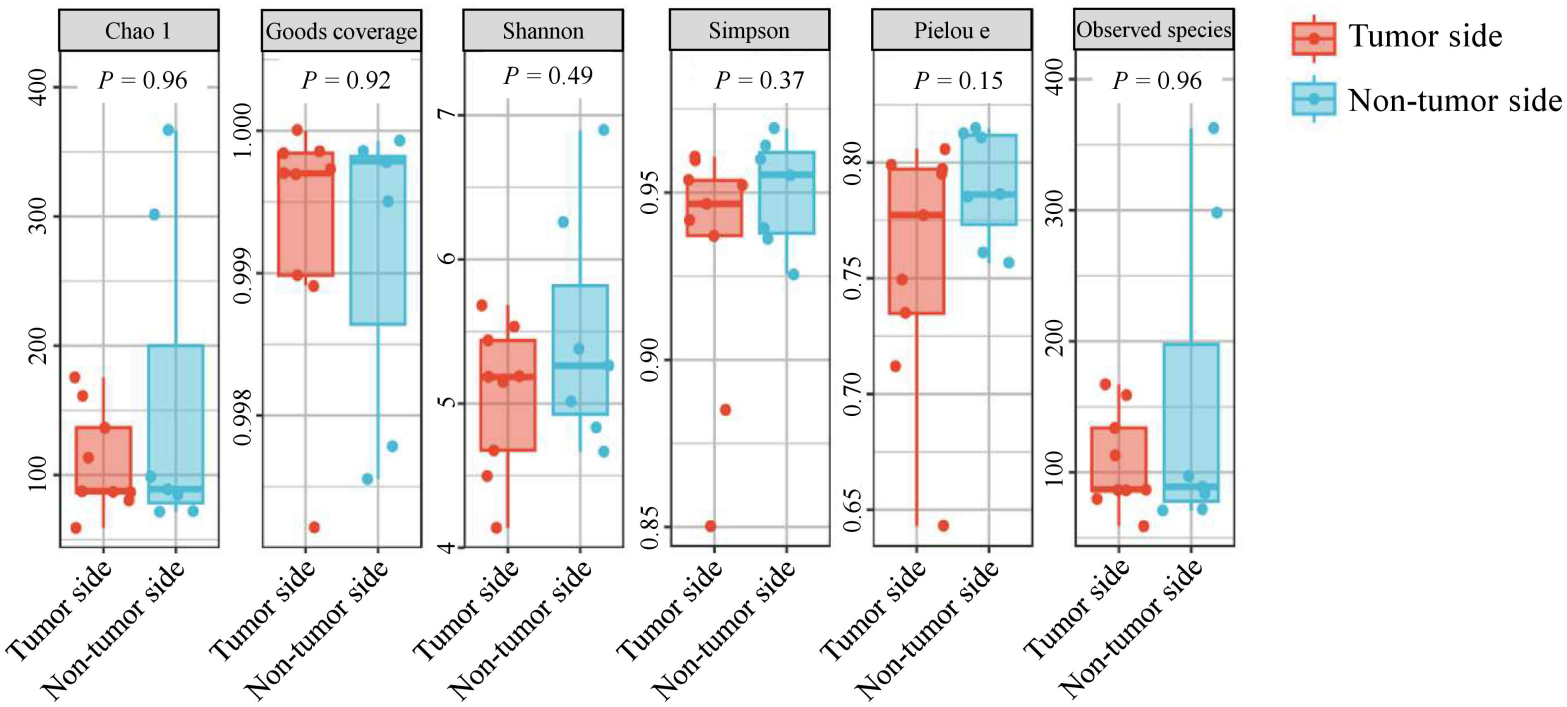
Alpha diversity analysis of microbial species in the oral cavity of the giant panda with an oral tumor.

The alpha diversity indexes Chao1 and observed species represent community richness, Shannon and Simpson indices represent community diversity, Pielou’s evenness index represents community evenness, and Good’s coverage represents the coverage of species in the community. In the tumor side and non-tumor side samples, the mean Chao1 indices were 109.70 and154.91, observed species indices were 107.93 and 153.39, Shannon indices were 5.05 and 5.47, Simpson indices were 0.93 and 0.95, and Pielou’s evenness indices were 0.76 and 0.79, respectively. However, the Good’s coverage was 99% for both samples, suggesting that the sequencing depth was sufficient to indicate the diversity of the samples (**Figure 2**). These results indicate no significant difference in the alpha diversity of the oral microbiota in the tumor side and non-tumor side of the giant panda.

### Beta diversity and group differences analysis

**Figure 3:**
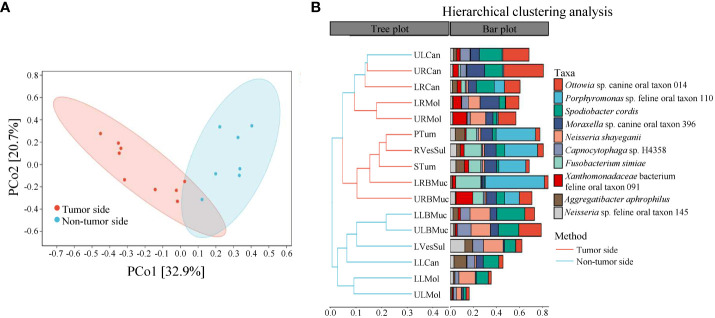
Beta diversity analysis of microbial species in the oral cavity of the giant panda with an oral tumor. **(A)** Principal coordinate analysis and **(B)** unweighted pair-group method with arithmetic means (UPGMA).

The beta diversity analysis was conducted to assess the similarity of microbial structure among different samples using the Bray-Curtis distance algorithm and PCoA analysis. The results revealed a difference between the community structure of the tumor side and non-tumor side samples, with the first two principal coordinate components accounting for 32.9% and 20.7% of the total variation, respectively (**Figure 3A**). The PERMANOVA analysis confirmed a significant difference between the tumor side and non-tumor side samples (*P* < 0.05). Similarly, the UPGMA analysis also demonstrated a notable difference between the microbial communities of the tumor side and non-tumor side samples (**Figure 3B**). Furthermore, the microorganisms on the teeth (including canine teeth and molars) as well as oral mucosa exhibited prominent clustering. Altogether, these results indicate a significant difference in the microbial composition of the tumor side and non-tumor side samples.

### Taxonomic analysis between the tumor side and non-tumor side

**Figure 4:**
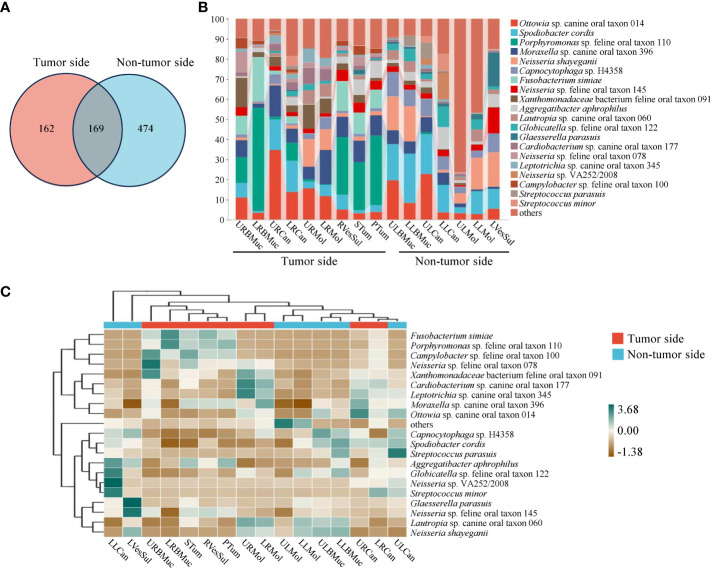
Analysis of species variations between the tumor side and non-tumor side samples of the oral cavity of the giant panda with an oral tumor. **(A)** Venn diagram analysis of amplicon sequence variants (ASVs) in the tumor side and non-tumor side samples. The quantities within the circles denote the number of ASVs in each group, while the quantity in the overlapping region denotes the number of shared ASVs between the two groups. **(B)** Species-level microbial composition of the tumor side and non-tumor side. **(C)** Heatmap of significant microbial clustering in the tumor side and non-tumor side at the species level.

The number of ASVs between the tumor side and non-tumor side samples was significantly different, with the tumor side containing 331 ASVs and non-tumor side containing 643 ASVs, among which 169 were common to both sides (**Figure 4A**). These results indicate that the microbial abundance is relatively low in the tumor side compared to the non-tumor side.

The top 20 most abundant species exhibited a distinct distribution pattern within the oral cavity of the giant panda. Specifically, the abundance of *F. simiae* (99.6%), *Porphyromonas* sp. feline oral taxon 110 (99.3%), *Campylobacter* sp. feline oral taxon 100 (98.9%), *Neisseria* sp. feline oral taxon 078 (98.3%), *Xanthomonadaceae* bacterium feline oral taxon 091 (86.4%), *Cardiobacterium* sp. canine oral taxon 177 (83.7%), and *Leptotrichia* sp. canine oral taxon 345 (82.0%) was significantly higher in the tumor side, with *F. simiae*, *Porphyromonas* sp. feline oral taxon 110, *Campylobacter* sp. feline oral taxon 100, and *Neisseria* sp. feline oral taxon 078 present exclusively in the tumor side of the oral cavity. In contrast, the abundance of *G. parasuis* (86.9%), *Neisseria* sp. VA252/2008 (86.1%), and *S. parasuis* (84.5%) was significantly higher in the non-tumor side of the oral cavity. The outer periphery of the tumor exhibited the highest abundance of *Porphyromonas* sp. feline oral taxon 110 and *F. simiae* (**Figure 4B**).

The cluster heatmap of the groups revealed a significant difference in microbial clustering between the tumor side and non-tumor side of the oral cavity (**Figure 4C**). The tumor side exhibited a more uniform microbiome compared to the non-tumor side, with *F. simiae*, *Porphyromonas* sp. feline oral taxon 110, *Campylobacter* sp. feline oral taxon 100, *Neisseria* sp. feline oral taxon 078, *Xanthomonadaceae* bacterium feline oral taxon 091, *Cardiobacterium* sp. canine oral taxon 177, and *Leptotrichia* sp. canine oral taxon 345 as the primary strains. Notably, *F. simiae*, *Porphyromonas* sp. feline oral taxon 110, *Campylobacter* sp. feline oral taxon 100, and *Neisseria* sp. feline oral taxon 078 were present exclusively in the tumor side. Furthermore, *N. shayeganii* was found to be present in low abundance in the mucosa surrounding the tumor, whereas it was significantly more abundant in the corresponding regions in the non-tumor side of the oral cavity (68.3%); however, the difference in the abundance of *N. shayeganii* in the dental plaques of tumor side (56.1%) and non-tumor side (43.9%) regions was not significant.

### Potential biomarkers

**Figure 5:**
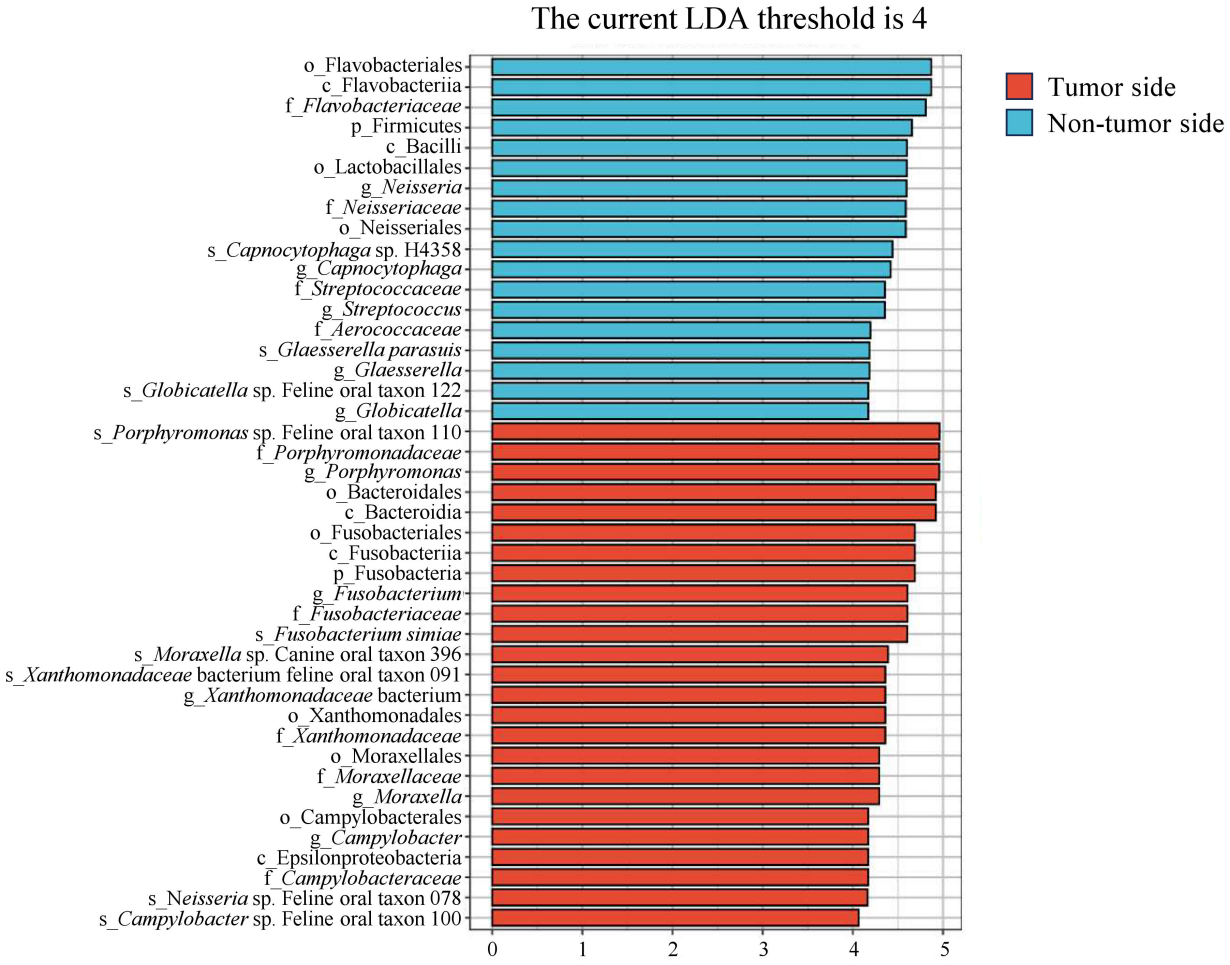
Linear discriminant analysis effect size analysis of microbial species in the tumor side and non-tumor side samples of the oral cavity of the giant panda with an oral tumor. The y-axis represents taxa exhibiting significant differences between the tumor side and non-tumor side samples, while the x-axis represents the score value of each taxon in the linear discriminant analysis. Taxa are hierarchically ranked based on their scores, which describe their specificity in grouping samples. Increased bar lengths signify more substantial differences for a given taxon, while the color scheme indicates the sample grouping corresponding to the highest abundance of that taxon.

After investigating the variations in microbial community composition, we determined the specific species in the tumor side and non-tumor side that contribute to these differences, using LEfSe analysis. We identified robustly differential species (marker species; *P* < 0.05) between the tumor side and non-tumor side samples, based on the linear discriminant analysis scores (> 4). The predominant marker species in the tumor side were *F. simiae*, *Porphyromonas* sp. feline oral taxon 110, *Xanthomonadaceae* bacterium feline oral taxon 091, *Moraxella* sp. canine oral taxon 396, *Campylobacter* sp. feline oral taxon 100, and *Neisseria* sp. feline oral taxon 078, while the predominant species in the non-tumor side were *Capnocytophaga* sp. H4358, *G. parasuis*, and *Globicatella* sp. Feline oral taxon 122, with *Streptococcus* being the primary genus (marker taxon; **Figure 5**). These results suggest that the marker species around the oral tumor were *F. simiae*, *Porphyromonas* sp. feline oral taxon 110, *Xanthomonadaceae* bacterium feline oral taxon 091, *Moraxella* sp. canine oral taxon 396, *Campylobacter* sp. feline oral taxon 100, and *Neisseria* sp. feline oral taxon 078. Among them, *F. simiae*, *Porphyromonas* sp. feline oral taxon 110, *Campylobacter* sp. feline oral taxon 100, and *Neisseria* sp. feline oral taxon 078 are consistent with the results of the taxonomic analysis conducted on the tumor side.

### Functional annotation

**Figure 6:**
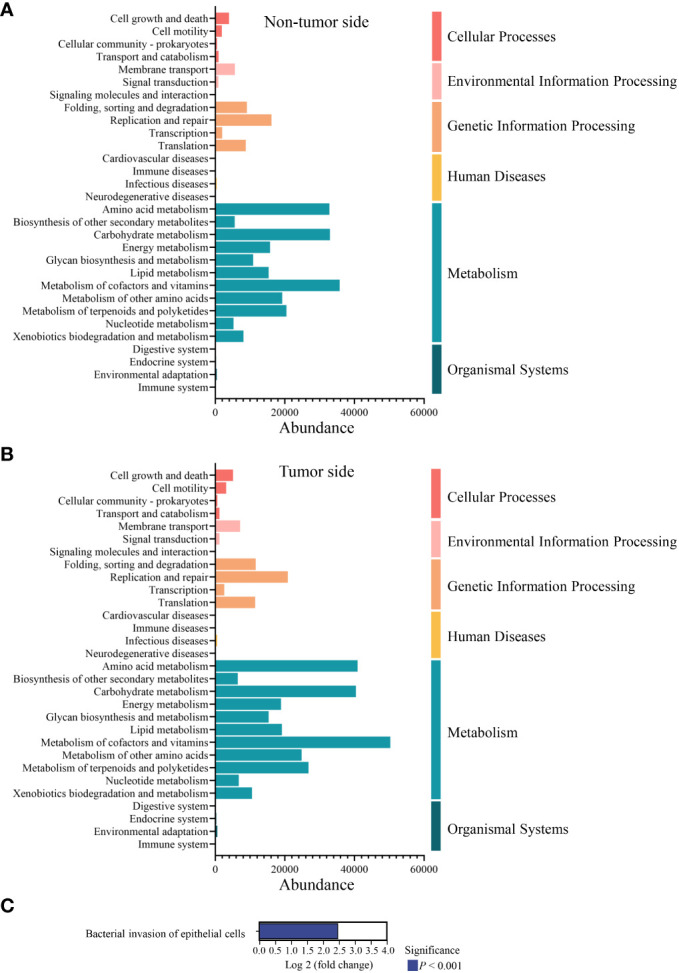
Metabolic pathway analysis of the abundant microbial species in the oral cavity of the giant panda with an oral tumor. **(A, B)** Relative abundance distribution of the second level based on Kyoto Encyclopedia of Genes and Genomes database. The x-axis represents the relative abundance of the species in the six functional groups, while the leftmost part of the y-axis indicates the functional pathway of the KEGG second classification level and the rightmost part of the y-axis indicates the first-level pathway to which the pathway belongs. The result of non-tumor side and tumor side are shown in panel **(A, B)**, respectively. **(C)** Comparison of the metabolic pathways associated with the abundant microbial species in the tumor side and non-tumor side of the oral cavity of the giant panda. The positive value of log 2 fold change signifies the up-regulation of the up-regulated group compared with the control group, with the ordinate representing different pathway tags.

KEGG metabolic pathway analysis revealed that the oral microbiota of the giant panda was predominantly associated with metabolism, especially cofactor and vitamin metabolism, amino acid metabolism, and carbohydrate metabolism, as well as genetic information processing, including replication and repair activities. The abundance of metabolic pathways was found to be higher on the tumor side compared to the non-tumor side; however, the overall metabolic pattern remained consistent on both sides. (**Figures 6A, B**). According to the KEGG orthologous group description of each metabolic pathway, we predicted that the metabolic disparity between the tumor side and non-tumor side may be the bacterial invasion of the epithelial cells (*P* < 0.001; **Figure 6C**).

## Discussion

The results of this study enhance our understanding of the association between tumorigenesis and the oral microbiome of giant pandas, providing information on its pathogenic mechanisms and preventative strategies. Advances in microbiome research have found that perturbations in the oral microbiome can hold significant implications for overall host health (Singhal et al., 2011). In this study, we used full-length 16S rRNA gene sequencing, a highly precise sequencing method, to conduct species-level identification of the oral microbiome of the giant panda with an oral tumor (Hall and Beiko, 2018; Johnson et al., 2019), thus enhancing the reliability of microbial diversity and abundance for pathological analysis.

In this study, we identified the oral microbiota composition and diversity in the tumor side and non-tumor side of the giant panda. We identified a total of 31 phyla, 55 classes, 89 orders, 136 families, 219 genera, and 402 species within the entire oral cavity. Diversity analysis revealed that the non-tumor side exhibited a higher microbial diversity compared to the tumor side, which may be attributed to the more uniform microbial ecosystem. These results are consistent with the previous oral microbiome studies in other animals (Adler et al., 2016; Tian et al., 2022). Additionally, a significant difference was observed in microbial clustering between the tumor side and non-tumor side, suggesting that the microbial composition on the two sides was distinct. Species richness on dental plaques was found to be notably higher than that on mucosa in both regions. This may be attributed to the complex composition of dental plaques, which facilitates microbial colonization (Velsko et al., 2019). This observation aligns with the concept that distinct regions within the oral cavity harbor unique microbial compositions, additionally, it is consistent with previous research demonstrating that oral mucosa exhibits lower richness and diversity while dental plaque showcases higher richness and diversity in humans (Xu et al., 2014; Zhang et al., 2018). However, this finding still required further investigation to establish its validity in the giant panda’s oral cavity.

The tumor side was dominated by specific microorganisms, including *F. simiae*, *Porphyromonas* sp. feline oral taxon 110, *Campylobacter* sp. feline oral taxon 100, and *Neisseria* sp. feline oral taxon 078. *F. simiae* was first isolated in 1982 from the dental plaque of a monkey (*Macaca arctoides*) (Slots and Potts, 1982). *Fusobacterium* sp. have been found to be associated with various tumorigenic processes in other organisms (Bullman et al., 2017; Fujiwara et al., 2020). *Porphyromonas gingivalis*, a member of *Porphyromonas* sp., is a well-studied periodontal pathogen (Reyes, 2021) that can promote tumor development by creating a carcinogenic microenvironment (Wen et al., 2020). Furthermore, *Campylobacter* and *Neisseria* species are known to cause various diseases, including diarrheal diseases, periodontitis, and other chronic conditions (Baral et al., 2007; Man, 2011), in both humans and animals (Liu et al., 2015). Therefore, variations in the abundance of these species may be associated with tumorigenesis or may be a consequence of tumor presence.

Furthermore, KEGG pathway analysis revealed that the oral microorganisms of the giant panda were predominantly associated with genetic information processing and metabolism, particularly cofactor and vitamin, amino acid, and carbohydrate metabolism, suggesting their crucial role in nutrient processing and host cellular processes. Moreover, a marked bacterial invasion of epithelial cells was predicted in the tumor side, indicating that the interplay between microbial communities and host tissues may contribute to the initiation and progression of tumors by inducing inflammatory responses, tissue damage, and creating a tumor-promoting microenvironment. However, further experimental investigations at morphological or molecular biology levels are required to substantiate this hypothesis.

In conclusion, this comprehensive investigation into the microbial dynamics in the giant panda with an oral tumor provides insights into microbial interactions and their potential impacts on health. The observed differences between the tumor side and non-tumor side, along with the identification of potential biomarkers, could facilitate further research into the oral health of giant pandas, thereby contributing to the improved care of captive and elderly giant pandas. These findings not only enrich our understanding of the oral health of giant pandas but could also hold broader implications for deciphering the relationships between the microbiome and diseases in other species.

## Data availability statement

The data presented in the study are deposited in the NCBI Sequence Read Archive (SRA) repository, accession number: PRJNA1053871.

## Ethics statement

All animal experiments were approved by Animal Care and Use Committee of the China Conservation and Research Center (Letter no: CCRCGP2020003). The study was conducted in accordance with the local legislation and institutional requirements.

## Author contributions

XW: Data curation, Formal analysis, Investigation, Methodology, Project administration, Software, Validation, Visualization, Writing – original draft, Writing – review & editing. MJ: Data curation, Formal analysis, Investigation, Methodology, Project administration, Software, Validation, Visualization, Writing – original draft, Writing – review & editing. QM: Data curation, Formal analysis, Investigation, Methodology, Project administration, Software, Validation, Visualization, Writing – original draft, Writing – review & editing. YWL: Data curation, Formal analysis, Investigation, Methodology, Project administration, Software, Validation, Visualization, Writing – original draft, Writing – review & editing. TZ: Data curation, Formal analysis, Investigation, Methodology, Project administration, Software, Validation, Visualization, Writing – original draft, Writing – review & editing. JY: Data curation, Formal analysis, Investigation, Methodology, Project administration, Software, Validation, Visualization, Writing – original draft, Writing – review & editing. LY: Conceptualization, Data curation, Formal analysis, Funding acquisition, Investigation, Methodology, Project administration, Resources, Software, Supervision, Validation, Visualization, Writing – original draft, Writing – review & editing. CW: Conceptualization, Data curation, Formal analysis, Funding acquisition, Investigation, Methodology, Project administration, Resources, Software, Supervision, Validation, Visualization, Writing – original draft, Writing – review & editing. YQL: Conceptualization, Data curation, Formal analysis, Funding acquisition, Investigation, Methodology, Project administration, Resources, Software, Supervision, Validation, Visualization, Writing – original draft, Writing – review & editing.

## Supplementary material

Supplementary Figure 1 Rarefaction curves of all the samples. The x-axis represents the depth of sampling, while the y-axis indicates the median value of the Chao1 index, calculated over 10 iterations.

Supplementary Figure 2 Length Distribution.
