# Supplementary material for: Oral microbiome sequencing revealed the enrichment of Fusobacterium sp., Porphyromonas sp., Campylobacter sp., and Neisseria sp. on the oral malignant fibroma surface of giant panda
Source: Front Cell Infect Microbiol. 2024 May 28;14:1356907. doi: 10.3389/fcimb.2024.1356907 (PMC11165184; doi:10.3389/fcimb.2024.1356907)
Supplement: Supplementary file 1 [file Table_1.docx]

| **Table S1. Relative abundance of the top 20 most abundant species in each sample.** |
| --- |

| **Name of species** | **Tumor side** | | | | | | | | |  | **Non-tumor side** | | | | | | |
| --- | --- | --- | --- | --- | --- | --- | --- | --- | --- | --- | --- | --- | --- | --- | --- | --- | --- |
|  | **URBMuc** | **LRBMuc** | **URCan** | **LRCan** | **URMol** | **LRMol** | **RVesSul** | **STum** | **PTum** |  | **ULBMuc** | **LLBMuc** | **ULCan** | **LLCan** | **ULMol** | **LLMol** | **LVesSul** |

| *Ottowia* sp. canine oral taxon 014 | 0.1100 | 0.0333 | 0.3464 | 0.1363 | 0.1546 | 0.1158 | 0.0496 | 0.0305 | 0.0390 |  | 0.1952 | 0.0837 | 0.2283 | 0.0366 | 0.0270 | 0.0247 | 0.0539 |
| --- | --- | --- | --- | --- | --- | --- | --- | --- | --- | --- | --- | --- | --- | --- | --- | --- | --- |
| *Spodiobacter cordis* | 0.0715 | 0.0087 | 0.1537 | 0.1568 | 0.0334 | 0.0540 | 0.0750 | 0.0173 | 0.0323 |  | 0.1793 | 0.2448 | 0.1961 | 0.1352 | 0.0242 | 0.1041 | 0.0983 |
| *Porphyromonas* sp. feline oral taxon 110 | 0.1285 | 0.5165 | 0.0117 | 0.0887 | 0.0114 | 0.0036 | 0.2873 | 0.2356 | 0.3432 |  | 0 | 0 | 0.0089 | 0.0011 | 0.0005 | 0.0014 | 0 |
| *Moraxella* sp. canine oral taxon 396 | 0.0862 | 0.0192 | 0.1563 | 0.0711 | 0.0632 | 0.1659 | 0.1009 | 0.1061 | 0.1022 |  | 0.0692 | 0.0599 | 0.0775 | 0.0608 | 0.0167 | 0.0086 | 0.0112 |
| *Neisseria shayeganii* | 0.0126 | 0.0047 | 0.0052 | 0.0160 | 0.1351 | 0.1029 | 0.0175 | 0.0204 | 0.0186 |  | 0.1690 | 0.1747 | 0.0035 | 0.0129 | 0.0428 | 0.1433 | 0.1719 |
| *Capnocytophaga* sp. H4358 | 0.0170 | 0.0069 | 0.0510 | 0.0094 | 0.0268 | 0.0577 | 0.0104 | 0.0138 | 0.0172 |  | 0.1210 | 0.0838 | 0.0870 | 0.0677 | 0.0266 | 0.0346 | 0.0934 |
| *Fusobacterium simiae* | 0.0909 | 0.2188 | 0.0176 | 0.0360 | 0.0042 | 0.0040 | 0.1503 | 0.1052 | 0.0915 |  | 0 | 0 | 0.0007 | 0.0003 | 0.0020 | 0 | 0.0003 |
| *Neisseria* sp. feline oral taxon 145 | 0.0431 | 0 | 0.0221 | 0.0201 | 0.0218 | 0.0167 | 0.0544 | 0.0492 | 0.0442 |  | 0.0298 | 0.0248 | 0.0281 | 0.0324 | 0.0057 | 0.0265 | 0.1272 |
| *Xanthomonadaceae* bacterium feline oral taxon 091 | 0.1462 | 0.0266 | 0.0466 | 0.0338 | 0.1201 | 0.0701 | 0.0330 | 0.0374 | 0.0118 |  | 0.0152 | 0.0208 | 0.0289 | 0.0048 | 0.0086 | 0.0015 | 0.0026 |
| *Aggregatibacter aphrophilus* | 0.0069 | 0.0208 | 0.0023 | 0.0413 | 0.0017 | 0.0096 | 0.0339 | 0.0745 | 0.0830 |  | 0.0159 | 0.0430 | 0.0287 | 0.1093 | 0.0117 | 0.0131 | 0.0664 |
| *Lautropia* sp. canine oral taxon 060 | 0.0018 | 0.0027 | 0.0155 | 0.0047 | 0.0697 | 0.0625 | 0.0140 | 0.0238 | 0.0058 |  | 0.0469 | 0.0520 | 0.0116 | 0.0007 | 0.0148 | 0.0551 | 0.0209 |
| *Globicatella* sp. feline oral taxon 122 | 0 | 0.0007 | 0.0106 | 0.0242 | 0.0286 | 0.0150 | 0.0134 | 0.0049 | 0.0063 |  | 0.0299 | 0.0610 | 0.0246 | 0.1003 | 0.0015 | 0.0446 | 0.0168 |
| *Glaesserella parasuis* | 0 | 0 | 0.0023 | 0.0058 | 0 | 0.0013 | 0 | 0.0223 | 0.0049 |  | 0.0127 | 0.0087 | 0.0119 | 0.0158 | 0.0009 | 0.0248 | 0.1691 |
| *Cardiobacterium* sp. canine oral taxon 177 | 0.0188 | 0.0016 | 0.0318 | 0.0384 | 0.0818 | 0.0559 | 0.0062 | 0.0193 | 0.0055 |  | 0.0079 | 0.0042 | 0.0141 | 0.0099 | 0.0087 | 0.0008 | 0.0049 |
| *Neisseria* sp. feline oral taxon 078 | 0.1026 | 0.0063 | 0.0066 | 0.0192 | 0.0274 | 0.0157 | 0.0137 | 0.0455 | 0.0144 |  | 0 | 0 | 0 | 0 | 0.0037 | 0.0006 | 0 |
| *Leptotrichia* sp. canine oral taxon 345 | 0.0163 | 0.0052 | 0.0369 | 0.0219 | 0.0675 | 0.0381 | 0.0058 | 0.0070 | 0.0016 |  | 0.0053 | 0.0039 | 0.0215 | 0.0101 | 0 | 0.0011 | 0.0020 |
| *Neisseria* sp. VA252/2008 | 0 | 0 | 0.0144 | 0.0100 | 0 | 0.0008 | 0 | 0.0009 | 0.0006 |  | 0 | 0 | 0.0288 | 0.1336 | 0.0020 | 0.0011 | 0.0004 |
| *Campylobacter* sp. feline oral taxon 100 | 0.0514 | 0.0187 | 0.0060 | 0.0147 | 0.0035 | 0.0025 | 0.0240 | 0.0443 | 0.0248 |  | 0 | 0.0002 | 0.0014 | 0.0004 | 0 | 0 | 0 |
| *Streptococcus parasuis* | 0.0026 | 0 | 0.0052 | 0.0179 | 0.0028 | 0 | 0.0002 | 0.0003 | 0.0003 |  | 0.0082 | 0.0501 | 0.0792 | 0.0110 | 0.0014 | 0.0035 | 0.0054 |
| *Streptococcus minor* | 0 | 0.0002 | 0.0009 | 0.0501 | 0 | 0 | 0.0005 | 0.0015 | 0.0009 |  | 0.0012 | 0.0014 | 0.0307 | 0.0826 | 0.0003 | 0 | 0.0048 |
| Others | 0.0935 | 0.1091 | 0.0569 | 0.1837 | 0.1422 | 0.1892 | 0.1100 | 0.1321 | 0.1453 |  | 0.0913 | 0.0826 | 0.0885 | 0.1747 | 0.6474 | 0.4289 | 0.1498 |
