# Supplementary figures and images for: Oral microbiome sequencing revealed the enrichment of Fusobacterium sp., Porphyromonas sp., Campylobacter sp., and Neisseria sp. on the oral malignant fibroma surface of giant panda
Source: Front Cell Infect Microbiol. 2024 May 28;14:1356907. doi: 10.3389/fcimb.2024.1356907 (PMC11165184; doi:10.3389/fcimb.2024.1356907)

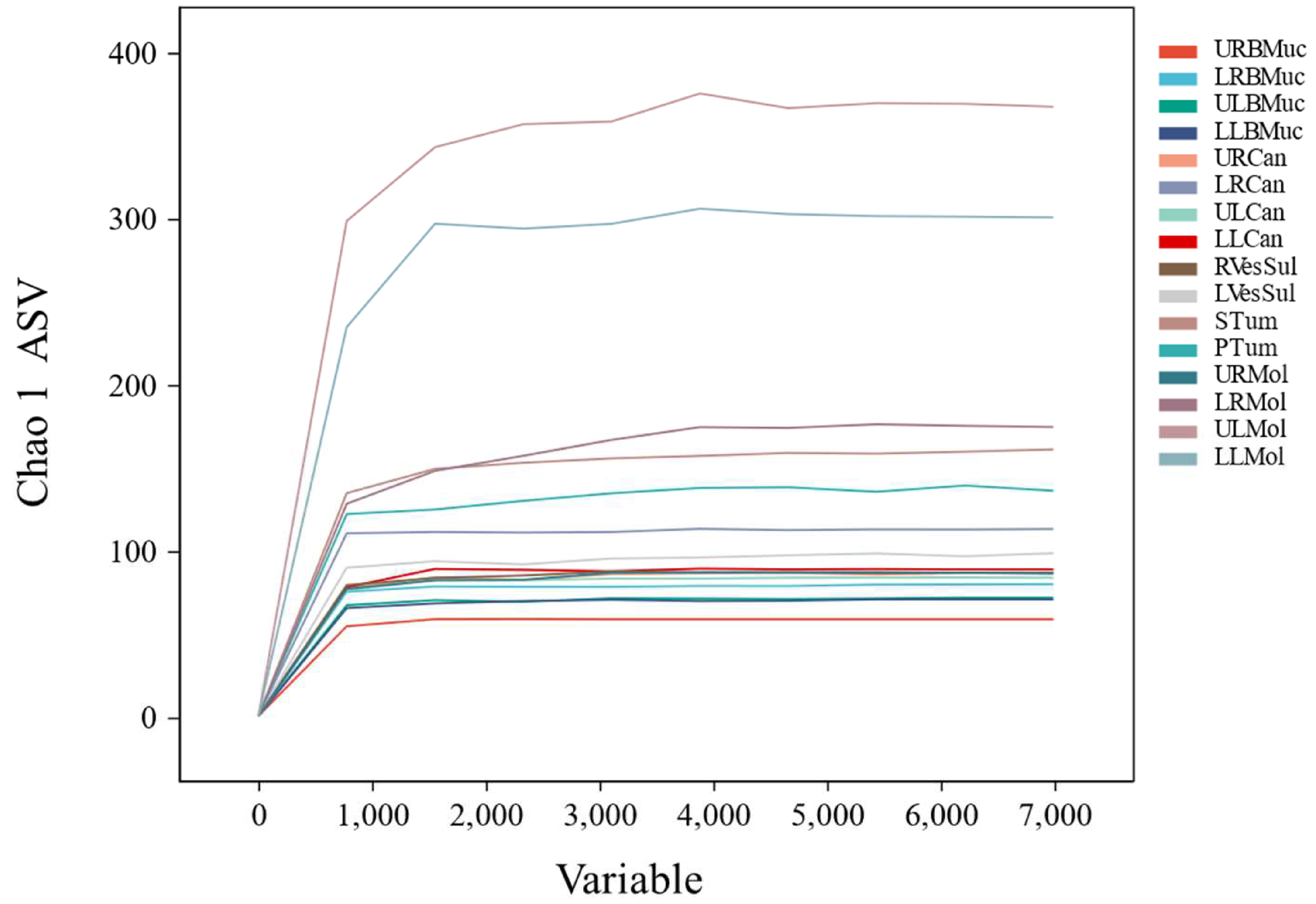

Supplement: Supplementary Figure 1 — Rarefaction curves of all the samples. The x-axis represents the depth of sampling, while the y-axis indicates the median value of the Chao1 index, calculated over 10 iterations. [file Image_1.tif]

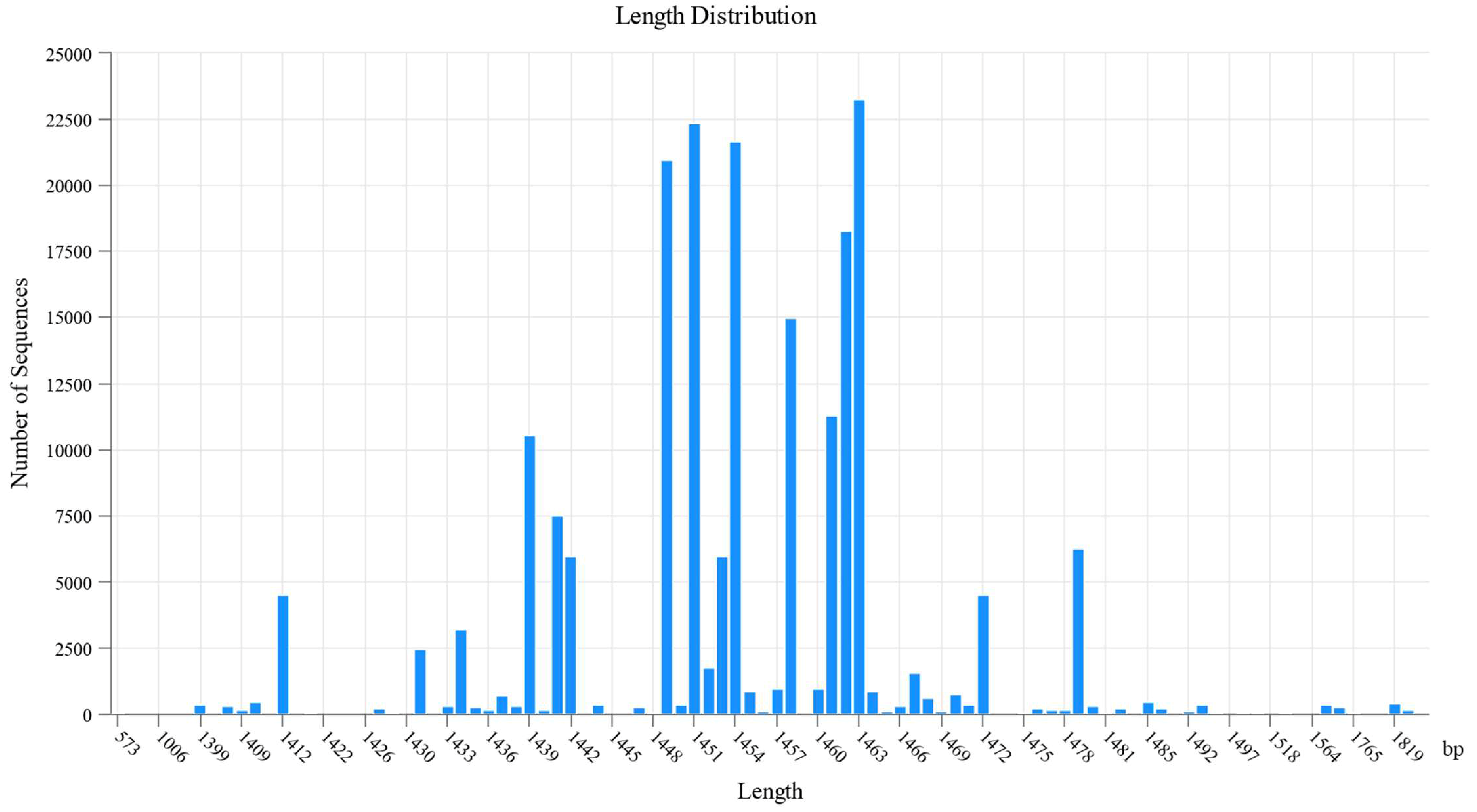

Supplement: Supplementary Figure 2 — Length Distribution. [file Image_2.tif]
